# A Novel Chimeric Endolysin with Antibacterial Activity against Methicillin-Resistant *Staphylococcus aureus*

**DOI:** 10.3389/fcimb.2017.00290

**Published:** 2017-06-30

**Authors:** Hamed Haddad Kashani, Hossein Fahimi, Yasaman Dasteh Goli, Rezvan Moniri

**Affiliations:** ^1^Anatomical Sciences Research Center, Kashan University of Medical SciencesKashan, Iran; ^2^Department of Molecular and Cellular Sciences, Faculty of Advanced Sciences and Technology, Pharmaceutical Sciences Branch, Islamic Azad UniversityTehran, Iran; ^3^Department of Biology, University of MarylandCollege Park, MD, United States

**Keywords:** *Staphylococcus aureus*, endolysin, antibacterial agent, CHAP-amidase domain, *in silico* analysis, antibiotic resistant, synergistic

## Abstract

Cysteine/histidine-dependent amidohydrolase/peptidase (CHAP) and amidase are known as catalytic domains of the bacteriophage-derived endolysin LysK and were previously reported to show lytic activity against methicillin-resistant *Staphylococcus aureus* (MRSA). In the current study, the *in silico* design and analysis of chimeric CHAP-amidase model was applied to enhance the stability and solubility of protein, which was achieved through improving the properties of primary, secondary and tertiary structures. The coding gene sequence of the chimeric CHAP-amidase was synthesized and subcloned into the pET-22(+) expression vector, and the recombinant protein was expressed in *E. coli* BL21 (DE3) strain. Subsequent affinity-based purification yielded ~12 mg soluble protein per liter of *E. coli* culture. Statistical analysis indicated that concentrations of ≥1 μg/mL of the purified protein have significant antibacterial activity against *S. aureus* MRSA_252_ cells. The engineered chimeric CHAP-amidase exhibited 3.2 log reduction of MRSA_252_ cell counts at the concentration of 10 μg/mL. A synergistic interaction between CHAP-amidase and vancomycin was detected by using checkerboard assay and calculating the fractional inhibitory concentration (FIC) index. This synergistic effect was shown by 8-fold reduction in the minimum inhibitory concentration of vancomycin. The chimeric CHAP-amidase displayed strong antibacterial activity against *S. aureus, S. epidermidis*, and *enterococcus*. However, it did not indicate any significant antibacterial activity against *E. coli* and *Lactococcus lactis*. Taken together, these findings suggest that our chimeric CHAP-amidase might represent potential to be used for the development of efficient antibacterial therapies targeting MRSA and certain Gram-positive bacteria.

## Introduction

Over the recent years, resistance to antimicrobial drugs has become a growing global concern. This health problem is rooted in the fact that antibiotic-based treatment of many infectious diseases has led to the emergence of multi-resistant strains of bacteria (Pogue et al., 2015). The rise of these multi-drug resistant bacterial strains highlights the need to develop new antimicrobial compounds (Magiorakos et al., 2012).

*Staphylococcus aureus* is known as one of the most significant infectious bacterial agents worldwide. The strains of this bacterium that have acquired methicillin resistance, also called MRSA, are considered a serious threat to human health (Reich et al., 2016).

Different species of the genus *Staphylococcus* are recognized as major pathogens for both human and various animals (De Lencastre et al., 2007). These bacteria are responsible for a wide variety of diseases, including skin and ocular infections, food poisoning, pneumonia, meningitis, endocarditis, and osteomyelitis (Nickerson et al., 2009; Bassetti et al., 2014). A considerable body of evidence has revealed that MRSA infection remains one of the main causes of hospital infections, leading to increasing rates of morbidity and mortality (Salge et al., 2016).

Bacteriophage endolysins are an important source of antimicrobial enzymes with peptidoglycan hydrolase activity. These antimicrobial agents, when applied exogenously in the form of purified recombinant proteins, can induce rapid lysis and death of Gram-positive bacterial cells (Jun et al., 2013). Phage-derived lysins represent potential use to target specific pathogenic bacteria, while not influencing the body's normal microbiota. A variety of investigations have discovered a broad range of lysins produced by different bacteriophages that can act specifically on certain host bacteria (O'flaherty et al., 2005; Hosseini et al., 2016; Schmelcher and Loessner, 2016). Previous studies have identified endopeptidase and amidase enzymes as potential therapeutics against a number of Gram-positive pathogens involved in triggering mucosal and systemic infections (Nelson et al., 2001; Schuch et al., 2002; Entenza et al., 2005; McCullers et al., 2007; Rashel et al., 2007; Daniel et al., 2010). As opposed to antibiotics, bacterial strains do not develop resistance to phage lysins (Loessner, 2005). These lysins target specific molecules in the host peptidoglycan that are required for cell viability (Fischetti, 2010). *Staphylococcus aureus* phage lysins, such as LysK, MV-L, phi11, and LysH5, possess a multi-domain structure composed of a C-terminal cell wall binding domain and two N-terminal catalytic domains (Donovan et al., 2006; Obeso et al., 2008). C-terminal domain has been reported to be required for lytic activities of endolysin (Loessner et al., 2002; Sass and Bierbaum, 2007). The majority of lysins bind to specific substrates in the bacterial cell wall through non-covalent carbohydrate bonds (Loessner et al., 2002; Nelson et al., 2006; Oliveira et al., 2016). The native lysins of *S. aureus*, except for MV-L, inherently display low expression yields, insolubility, and poor activity when generated *in vitro* (Fenton et al., 2010).

A number of studies have demonstrated the increased lytic activity of several enzymes upon deletion of their binding domains (Cheng et al., 1994; Cheng and Fischetti, 2007; Horgan et al., 2009). Compared with native enzyme, the truncated N-terminal endopeptidase domain, also known as CHAP (cysteine, histidine-dependent amidohydrolase/peptidase), of LysK indicates significantly higher levels of antibacterial lytic activity (Horgan et al., 2009). Once the C-terminal binding domain of native enzyme is bound to its target molecule in the bacterial cell wall, the function of N-terminal lytic domain may be altered, leading to inhibition of the potential activity of lytic domain (Low et al., 2005; Briers et al., 2009). LysK proteins containing CHAP and amidase domains have been reported to exhibit lytic activity against MRSA strains (Becker et al., 2009). Previous studies have shown that the recombinant production of LysK protein in *E. coli* may result in the formation of insoluble inclusion bodies, possibly due to the adverse formation of disulfide bonds in the protein (Ventura and Villaverde, 2006; Yoon et al., 2010). Proteolytic degradation and protein misfolding are common problems in the expression of recombinant proteins in bacterial cells. These difficulties are frequently encountered, especially if the protein is insoluble and cytoplasmic. To surmount such obstacles, recombinant proteins can be targeted to the periplasmic space via the activity of signal sequences like PelB (Low et al., 2013). It is also of interest to note that formation of inclusion bodies in *E. coli* seems to be a universal problem associated with overexpression of heterologous proteins (Rinas et al., 2007; Martínez-Alonso et al., 2009).

pET-22b is a family of pET vectors providing an N-terminal pelB leader signal, which directs the secretion of expressed polypeptides to the *E. coli* periplasmic space (Yoon et al., 2010). The periplasmic space of *E. coli* cells harbors oxidoreductive enzymes, which play important roles in preventing the formation of disulfide bonds and inclusion bodies (Berkmen, 2012). The *in silico* analysis of CHAP-amidase has provided valuable insights into the structure and function of lysins (Fenton et al., 2011a). This information can be used for the design and selection of essential parts of CHAP-amidase to assess its antibacterial activity against MRSA_252_ strain.

Here, we report the *in silico* design and analysis, expression, purification, and subsequent characterization of the antibacterial activity of a chimeric endolysin against MRSA_252_ bacterial cells. We evaluated the activity of chimeric endolysin in various conditions and observed its synergistic activity when combined with vancomycin. In addition, we examined the antibacterial effects of chimeric CHAP-amidase against other bacterial strains, including *S. aureus, S. epidermidis, E. faecium, E. faecalis, E. coli*, and *L. lactis*.

## Materials and methods

### Construction and analysis of chimeric CHAP-amidase

Prior to synthesis of the sequence encoding chimeric CHAP-amidase, the sequence was optimized by Gen Script codon optimization software (http://www.genscript.com/).

The sequence of LysK was obtained from GenBank database (Accession No: AFN3871, www.ncbi.nlm.nih.gov/genbank/). The chimeric protein consists of the N-terminal CHAP domain (amino acids 38–164) fused to the C-terminal amidase-2 domain (amino acids 210–334) of LysK (Supplementary Figure 1). The two domains were connected by a decapeptide linker (GSHHHHHHGS), which also served as an internal His-tag for affinity purification. The designed amino acid sequence was analyzed using the Basic Local Alignment Search Tool (BLAST) (http://blast.ncbi.nlm.nih.gov/Blast.cgi). The Protparam (http://web.expasy.org/protparam/) and HMMTOP servers (www.enzim.hu/hmmtop) were used to investigate the primary structure properties and to predict trans-membrane helices, respectively. Secondary structures were predicted using Chou and Fasman method (http://cho-fas.sourceforge.net/index.php). Tertiary structures were modeled in PDB format via I-TASSER server (Zhang, 2008; Roy et al., 2010; Yang and Zhang, 2015; Yang et al., 2015). The constructed structural models were analyzed by PyMol software. For model stability studies, 3D PDB models were subjected to evaluation by Ramachandran plot analysis (RAMPAGE software) (Lovell et al., 2003) and PDBsum server (http://www.ebi.ac.uk/thornton-srv/databases/pdbsum).

### Docking of protein-ligand binding

The interaction of CHAP-amidase protein with cysteine, glycerol and piperazine ligands was analyzed by HEX 6.3 software, which allows to predict the free energy of binding (Ritchie and Venkatraman, 2010).

### Design and synthesis of the coding gene fragment

The gene sequence was optimized in terms of codon usage and GC content for optimal expression in *E. coli* by using the web-based server optimizer (http://genomes.urv.cat/OPTIMIZER/). The restriction sites for *HindIII* and *NcoI* enzymes were located at 3′ and 5′ sites of the coding sequence, respectively. The designed gene was synthesized by MWG-Biotech (Ebersberg, Germany) and incorporated into a pEX backbone.

### Bacterial culture, subcloning, and protein expression

The *E. coli* strain BL21 (DE3) (Invitrogen, Carlsbad, CA) was grown at 37°C in Luria-Bertani (LB) medium (Rosano and Ceccarelli, 2014). The LB medium was then supplemented with ampicillin (100 μg/mL) for plasmid selection. The subcloning of CHAP-amidase-encoding sequence into plasmid and transformation of pEX and pET-22b (Novagen, USA) vectors into bacterial cells were followed by standard restriction enzyme digestion and sequence analysis, which were used to verify the cloning procedure.

Protein expression was performed in BL21 (DE3) strain as previously reported (Kashani and Moniri, 2015; Hosseini et al., 2016). Briefly, the recombinant pET-22b plasmid was transformed into bacterial cells and the cells were cultured in LB medium containing 100 μg/mL of ampicillin. The culture was further incubated at 37°C and protein expression was induced by addition of isopropyl β-D-1-thiogalactopyranoside (IPTG) to the final concentration of 1 mM at logarithmic phase (corresponding to 0.5–0.6 OD_590_). Cells were harvested after 4 h and protein expression was evaluated by SDS-PAGE (sodium-dodecyl sulfate polyacrylamide gel electrophoresis).

### Western blot analysis

Western blot analysis was conducted as previously reported (Fahimi et al., 2014; Ferdosian et al., 2015; Hosseini et al., 2016). Briefly, the lysate of CHAP-amidase-expressing cells was run on 10% SDS-PAGE (Bioneer Co., South Korea) and transferred onto nitrocellulose membrane using a semi-dry transfer system. The membrane was blocked overnight in 5% skimmed milk at 4°C and then incubated with primary antibody (anti-His tag monoclonal antibody (mAb) (Abcam Inc., MA, USA), while shaking for 2 h at room temperature (22°C). The membrane was washed three times with PBST (PBS containing 0.05% Tween 20) and incubated with secondary antibody (1:4,000 dilution of HRP-conjugated rabbit anti-mouse IgG antibody) (Abcam Inc., MA, USA) with gentle shaking for 1 h at room temperature. The membrane was washed three times with PBST and the signal was detected by adding DAB (3,3′-diaminobenzidine) as a chromogenic substrate.

### Protein purification

The recombinant expression cultures of BL21 were harvested by centrifugation and the pellets were lysed via sonication. By using the modified nickel-chromatography Ni-NTA purification system (Qiagen, Valencia, CA), his-tagged proteins were separated according to the manufacturer's instructions. Briefly, Ni-NTA column was washed in two consecutive steps with 20 mL/20 mM and 10 mL/10 mM and then eluted with 0.5 mL of 250 mM imidazole in the same phosphate-buffered saline (PBS) (300 mM NaCl, 50 mM NaH2PO3, pH 8) using 30% glycerol. Then, the samples were transferred into a buffer with low salinity (10 mM Tris-HCl, 150 mM NaCl, 1% glycerol; pH 7.5) by Zeba Desalting column (Thermo Fisher Scientific Inc., USA) and sterilized with 0.22 μm filters and equilibrated in 2X PBS buffer. The concentration of purified protein determined by spectrophotometry using the Bradford assay (Achberger et al., 2011).

### Characterization of antibacterial activity against methicillin-resistant *S. aureus*

The lytic effects of CHAP-amidase were investigated on methicillin-resistant *Staphylococcus aureus* strain (MRSA_252_). The bacterial strain was purchased from ATCC and cultured in mannitol salt agar (MSA) medium.

#### Disk diffusion assay

The purified protein was diluted in saline Tris lysis buffer (STB), containing 150 mM NaCl and 10 mM Tris-HCl (pH 7.5), to the final concentrations of 10, 1, 0.5, and 0.1 mg/mL. Afterwards, a sterile 6 mm filter paper disk was used per concentration of CHAP-amidase. The saturated disks were spotted onto a freshly spread MRSA culture, while control disk was soaked in STB (Kusuma and Kokai-Kun, 2005; Becker et al., 2009; Jalali et al., 2016). The culture was incubated for 20 h at 37°C.

#### Specific activity of CHAP-amidase endolysin

To study the thermal stability of CHAP-amidase, the protein was pretreated under a range of temperatures from 5 to 60°C. The protein stability was also evaluated at −20°C, 4°C, and 37°C. The specific activity of CHAP-amidase against MRSA_252_ was determined by spectrophotometry (Eppendorf, Germany) at wavelength of 590 nm for 15 min and the number of bacterial colonies were evaluated by colony count assay (Fenton et al., 2011b).

To evaluate the specific activity of CHAP-amidase in different pH values, MRSA_252_ cells were resuspended in sodium acetate buffer (50 mmol/L) with pH values ranging from 4 to 12. 100 μL (1 μg/mL) of CHAP-amidase was added to cells (8 × 10^8^ CFU/mL) and OD_590nm_ was monitored at 37°C for 15 min (Fenton et al., 2011b). The final enzyme concentration in each sample was 1 μg/mL.

The tolerability of CHAP-amidase in different concentrations of NaCl was measured in mid-log phase cultures (OD_590nm_ ~ 0.6) of MRSA_252_ cells. Sodium acetate buffer (50 mmol/L; pH 7.5) was prepared containing various concentrations of NaCl from 0 to 700 mmol/L. 100 μL of cell solution incubated with 100 μl of CHAP-amidase (10 μg/mL) was examined at 590 nm for 30 min and finally changes in colony forming unit (CFU) values were monitored.

#### Antibacterial activity of different concentrations of endolysin on MRSA

By growing mid-log phase cultures (OD_590nm_ ~ 0.5–0.6) in Mueller-Hinton broth (MHB) (37°C), freshly grown MRSA_252_ cells were obtained and then harvested by centrifugation (4°C). Cells were recovered using 1/2 volumes of a phosphate buffer, containing 250 mM NaCl and 25 mM phosphate-Na (pH 7.5). Subsequently, various concentrations of chimeric CHAP-amidase (10, 5, 1, and 0.5, μg/mL) were added to 1 mL cell suspensions. OD_590nm_ was recorded for 1 h with 5-min intervals. The colony forming unit (CFU/mL) was calculated at the end of each assay. The buffer without endolysin was used as negative control (Becker et al., 2009; Fernandes et al., 2012; Proença et al., 2012).

#### Synergistic interaction of CHAP-amidase with vancomycin

The interaction between CHAP-amidase and vancomycin (Sigma-Aldrich) was assessed by standard checkerboard broth microdilution assay (Odds, 2003; Anju et al., 2015; Magi et al., 2015). Briefly, a 2-fold dilution of antibiotic and CHAP-amidase (in a final volume of 50 μL) was incubated vertically and horizontally, respectively, with a bacterial inoculum of 5 × 10^5^ CFU per well. MRSA_252_ strain was used to test the interaction between endolysin and vancomycin. The plates were incubated at 37°C with gentle shaking and the bacterial growth rate was determined by reading OD_590nm_ for 20 h. The fractional inhibitory concentration (FIC) of antibiotic and CHAP-amidase was calculated and plotted as an isobologram. The calculation of FIC index (Σ) was performed according to the following formula: FIC = MIC (in combination with CHAP-amidase)/MIC (alone) = > (FICA = MICA+B/MICA, FICB = MICB+A/MICB, ΣFIC = FICA+FICB). Synergism is defined as a ΣFIC of ≤0.5.

#### Lytic activity of CHAP-amidase endolysin against other bacterial strains

The activity of the three isolates of methicillin-resistant *Staphylococcus*, one isolate of methicillin-susceptible *Staphylococcus*, two isolates of vancomycin-resistant *Enterococcus*, one isolate of multi-drug resistant *E. coli*, and also *Lactococcus lactis* as a well-known genus of probiotic bacteria was measured in response to the chimeric endolysin by turbidity reduction assay and colony counting (Table 1). 10 μg/mL of CHAP-amidase was added to different microtubes of bacterial cultures and incubated at 37°C for 15 min. Colony count assay was carried out to determine whether turbidity reduction is due to bacterial cell death.

**Table 1:** Bacterial species evaluated for sensitivity to CHAP-amidase.

**Species**	**Strain**	**Description**	**Isolation**
*Staphylococcus aureus*	MRSA_252_	Methicillin resistant (ATCC BAA-1720)	Hospital acquired
*Staphylococcus aureus*	USA_300_	Methicillin resistant (ATCC BAA-1556)	HIV+ Patient
*Staphylococcus aureus*	MSSA_476_	Methicillin susceptible (ATCC BAA-1721)	Hyper virulent community
*Staphylococcus epidermidis*	CCF_15990_	Methicillin resistant (ATCC 51625)	Human blood
*Entrococcus faecalis*	AGR_329_	Vancomycin resistant (ATCC BAA-2128)	Piglet feces
*Entrococcus faecium*	–	Vancomycin resistant (ATCC BAA-2317)	Human feces
*Escherichia coli*	NDM_1_	Multi-drug resistant (ATCC BAA-2452)	Pakistan
*Lactococcus Lactis*	NZ_9000_	Probiotic (VS-ELS09000-01)	MoBitec, Germany

### Statistical analysis

One-way analysis of variance (ANOVA) and Tukey's *post-hoc* test were employed to study differences between the five groups. *P* < 0.05 were considered statistically significant. All statistical analyses were done using the Statistical Package for Social Science version 19 (SPSS Inc., Chicago, Illinois, USA). The values of experiments are the means of three independent experiments with indication of standard deviation (SD) (Kashani et al., 2013; Sharif et al., 2016, 2017).

## Results

### *In silico* analysis of LysK (CHAP-amidase)

#### Codon optimization

The codon adaptation index (CAI) of chimeric CHAP-amidase was initially 0.68, which is considered a weak expression index, but reached 0.88 after being optimized by Gen Scripts Optimum Gene software. There is a positive correlation between the possibility of high protein expression level and the value of CAI. A CAI of 1.0 is described as ideal and a CAI of ≥0.8 is considered as appropriate for expression in the host organism. Moreover, GC content was first 36.11%, which was changed into 52.33% following optimization (Supplementary Figure 2). It means that the codon usage of the gene is required to have a relatively higher GC content so that chimeric LysK could be expressed well in BL21 strain.

#### Sequence analysis of CHAP-amidase protein

**Figure 1:**
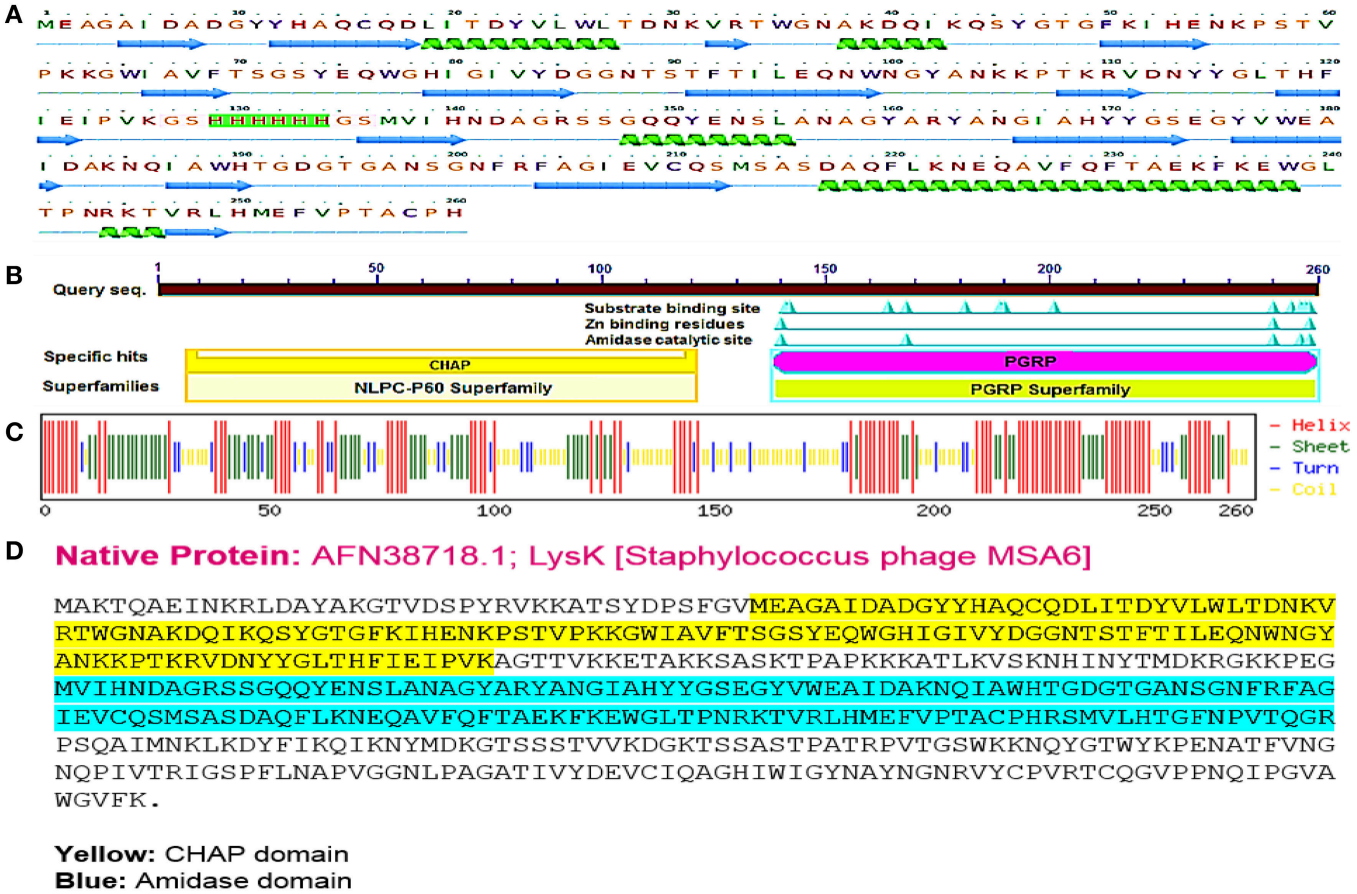
The designed “CHAP-amidase” protein properties. **(A)** The amino acid sequence of the designed LysK/CHAP-amidase protein consists of N-terminal CHAP domain, GS-His tag linker, and C-terminal amidase domain. The linker sequence is highlighted in pink for GS and green for 6x-His tag. The secondary structure of CHAP-amidase protein was analyzed by Phyre2 software and is displayed below the sequence as alpha helix (18%) and beta strand (32%) structures (Kelley et al., 2015). **(B)** According to the BLAST analysis of the LysK amino acid sequence, two catalytic CHAP (yellow) and amidase (purple) domains were recognized. **(C)** The predicted secondary structure of CHAP-amidase protein; the statistics are as follows: total residues: H (helix): 137, E (beta sheet): 89, T (turn): 37, and the frequency of structures: H: 52.7, E: 34.2, T: 14.2. **(D)** The native amino acid sequence of LysK containing 495 amino acids.

It has been revealed that CHAP and amidase [peptidoglycan recognition protein (PGRP) superfamily] domains show antibacterial activity against *Staphylococcus aureus* (Becker et al., 2009; Horgan et al., 2009). The sequence of CHAP-amidase protein starts from an N-terminal domain of CHAP (amino acids 38–164 of the original sequence) that is fused to amidase domain (amino acids 210–334 of the original sequence) via a 6x-His tag flanked by glycine and serine (GSHHHHHHGS). The amino acid sequence of chimeric protein starts with methionine and ends with histidine as shown in Figures 1A,D. The BLAST analysis of the designed amino acid sequence led to the recognition of two catalytic CHAP and amidase domains, which are members of NLPC/P60 and PGRP super families, respectively (Figure 1B).

According to the primary structure analysis of CHAP-amidase fusion protein, isoelectric point (pI) and instability index (II) were computed 6.73 and 30.94, respectively. These computations identify CAHP-amidase fusion as a stable protein in bacteria. No transmembrane helix was detected by HMMTOP server. The protein secondary structure was predicted by Chou and Fasman method. As depicted in Figure 1C, CHAP-amidase protein consists of mostly alpha-helix and beta-sheet regions separated by turns and coils. The designed linker introduces a coil into the structure (residues 127–136).

**Figure 2:**
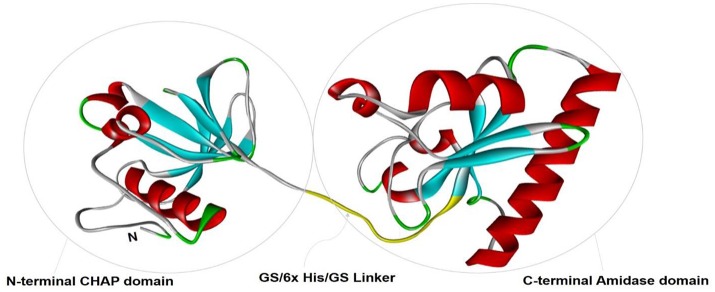
The tertiary structural model of CHAP-amidase protein was visualized by Pymol software. Two functional and structural domains (CHAP and amidase domains) were predicted for independent folding. These domains were linked together via a GS/6xHis/GS sequence.

To study the tertiary structure of CHAP-amidase protein, I-TASSER server was used to construct 3-dimensional structural models in PDB format. As illustrated in Figure 2, functional and structural domains were predicted to be folded independently and linked together via a GS/6xHis/GS linker.

**Figure 3:**
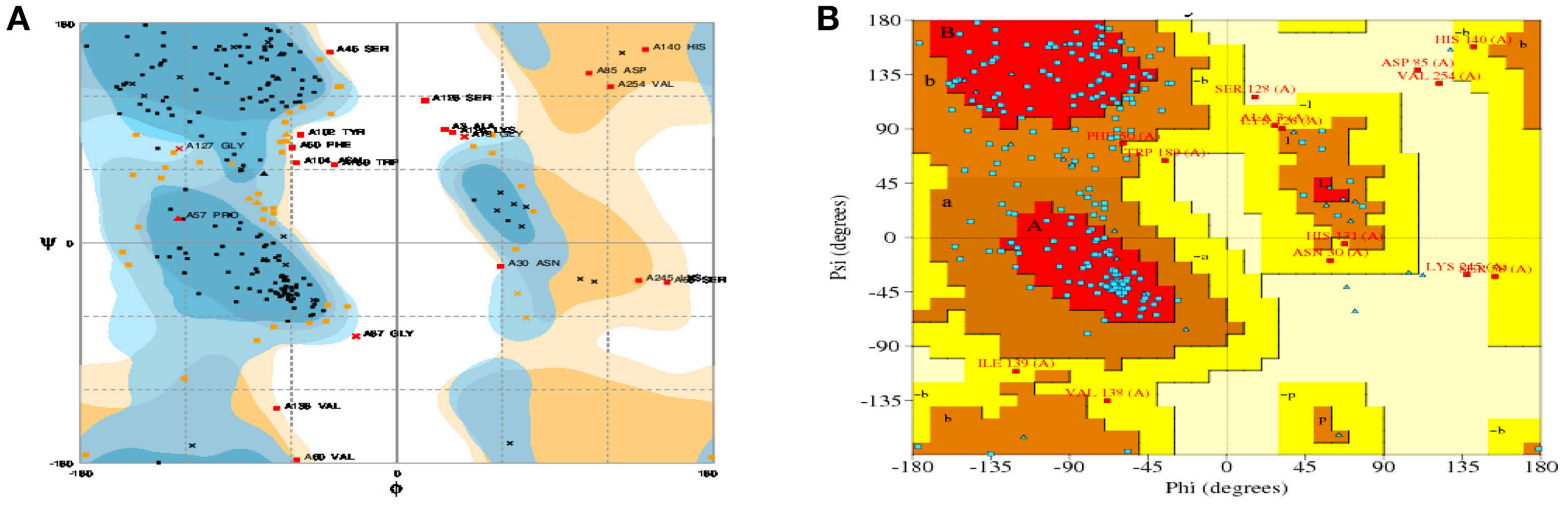
Ramachandran plots for the predicted 3D structure of CHAP-amidase created by RAMPAGE **(A)** and PDBsum **(B)**. Plot statistics indicate the number of residues in favored regions: 192 (74.4%), the number of residues in allowed regions: 46 (17.8%), and the number of residues in outlier regions: 20 (7.8%).

For model stability studies, 3D models were examined by Ramachandran plot analysis (Figure 3). More than 90% of residues are identified in most favored and sterically-allowed regions. These features reflect the high sustainability of constructed models.

#### Molecular docking of chimeric CHAP-amidase

**Figure 4:**
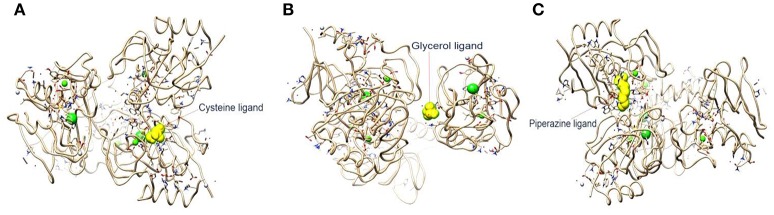
**(A)** 4ct3/cysteine binding energy = −150.67 kJ/mol. **(B)** 4ct3/glycerol binding energy = −129.83 kJ/mol. **(C)** 4ct3/piperazine binding energy = −228.66 kJ/mol.

In the current study, the binding energy of cysteine, glycerol and piperazine to the protein (PDB accession code 4ct3) was estimated to be −150.67 kJ/mol, −129.83 kJ/mol, and −228.66 kJ/mol, respectively (Figure 4). This finding suggests that interaction between CHAP-amidase and piperazine may be more potent than that of other ligands.

### Subcloning of the synthesized gene sequence

**Figure 5:**
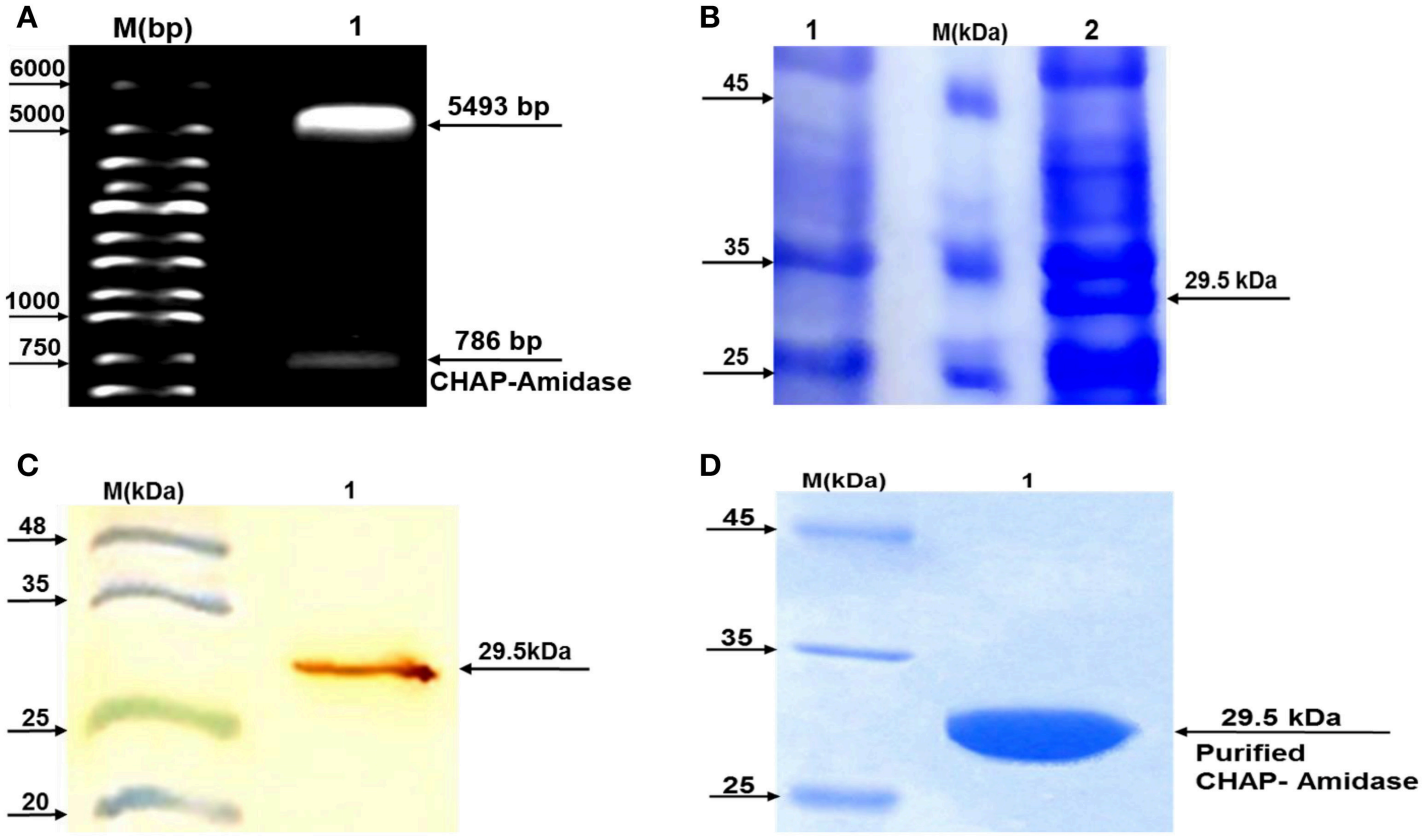
Analysis of LysK-CHAP sequence by agarose gel electrophoresis and analysis of protein expression by SDS-PAGE and western blotting. **(A)** Lane 1: pET-22b/CHAP-amidase plasmid double digested by *NcoI* and *HindIII* restriction enzymes. Lane M: Molecular size marker (1 kb ladder). Arrows indicate the position of DNA fragments. The presence of a 786 bp fragment confirms the accuracy of the cloning of LysK-b/CHAP-amidase gene into pET-22 vector. **(B)** Expression analysis of CHAP-amidase derived from the soluble phase of *E. coli* cell lysate, by SDS-PAGE (10%). Lane 1: Bacterial lysate before induction by IPTG, M: Molecular weight markers (Fermentas Company), Lane 2: Bacterial lysate after induction by IPTG. **(C)** Western blot analysis of the expressed CHAP-amidase protein reacted with anti-His tag mAb. M: Pre-stained protein molecular weight markers, Lane 1: CHAP-amidase-expressing bacterial lysate. The arrow indicates the position of the overexpressed CHAP-amidase recombinant protein. **(D)** Purification of CHAP-amidase; M: Protein marker, 1: Purified protein.

Subcloning of the sequence encoding CHAP-amidase was analyzed first by double digestion of pET-22 vector and running DNA fragments on agarose gel. The detection of 786 bp band confirmed accuracy of the cloning of CHAP-amidase gene into the vector (Figure 5A). Finally, the construct was sequenced (GenBank accession number: BankIt2010168 Chimeric KY967619).

### Protein expression

To analyze protein expression, a typical induction experiment was conducted based on comparing the protein profiles of cell lysates obtained from non-induced and induced bacterial cultures (Figure 5B). The results indicated that IPTG can induce the desired expression of CHAP-amidase (~29.5 kDa). The overexpressed recombinant protein was identified in the lysate by western blot analysis (Figure 5C).

### Purification of CHAP-amidase protein

The detection of protein band (~29.5 kDa) confirmed the purification of CHAP-amidase (Figure 5D). Our procedure led to the purification of 8–12 mg/L of protein in induced *E. coli* culture.

### Lytic activity of purified CHAP-amidase

Our results demonstrated that the purified CHAP-amidase has antibacterial activity against MRSA_252_. The inhibition zone of MRSA_252_ growth was detected by disks saturated with different concentrations of CHAP-amidase (1, 0.5, and 0.1 μg/μL). This observation was in accordance with the lytic activity of standard purified protein. However, a significant halo, which is caused by bacterial lysis, was not observed at the concentration of 0.05 μg/μL (Supplementary Figure 3).

### Thermal and pH stability

**Figure 6:**
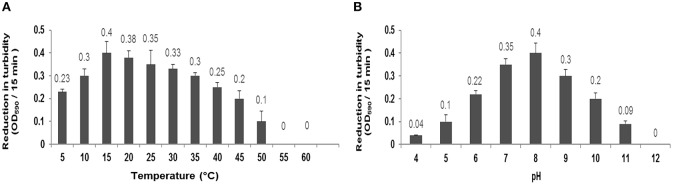
**(A)** Characterization of CHAP-amidase protein (1 μg/mL) in different temperatures ranging from 5 to 60°C. **(B)** Characterization of CHAP-amidase protein in different pH valus ranging from 4 to 12. Data are expressed as the mean ± SD.

There was not observed any significant alteration in the activity of CHAP-amidase under different temperatures, ranging from 5 to 60°C, for 15 min. Our findings indicated that the ideal activity of lysin occurs at 15–20°C (Figure 6A). During 40-h incubation at 37°C, the protein retained its activity; however, it was deactivated after 2 days. The lysin protein remained active for 1 month and 1 year when stored at 4°C and −20°C, respectively.

The specific activity of MRSA_252_, incubated with 1 μg/mL of endolysin, was monitored in different pH values for 15 min by spectrophotometry. The optimum activity of CHAP-amidase was at pH 8; but the protein also exhibited favorable activity at pH values from 6 to 10 (Figure 6B).

### NaCl tolerance

**Figure 7:**
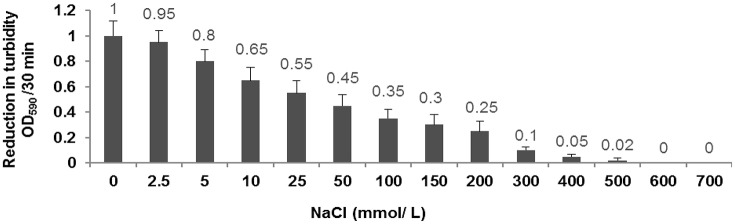
Characterization of the activity of 10 μg/mL CHAP-amidase in different NaCl concentrations ranging from 0 to 700 mmol/L. Data are expressed as the mean ± SD.

Our results demonstrated that decreasing the concentration of NaCl from 200 to 0 mmol/L leads to the increase of the enzymatic activity of CHAP-amidase protein. In 300–500 mmol/L concentrations of NaCl, the enzymatic activity was considerably reduced and no significant activity of CHAP-amidase was detected in 600–700 mmol/L concentrations (Figure 7).

### Effects of different concentration of CHAP-amidase on MRSA_252_ cells

**Figure 8:**
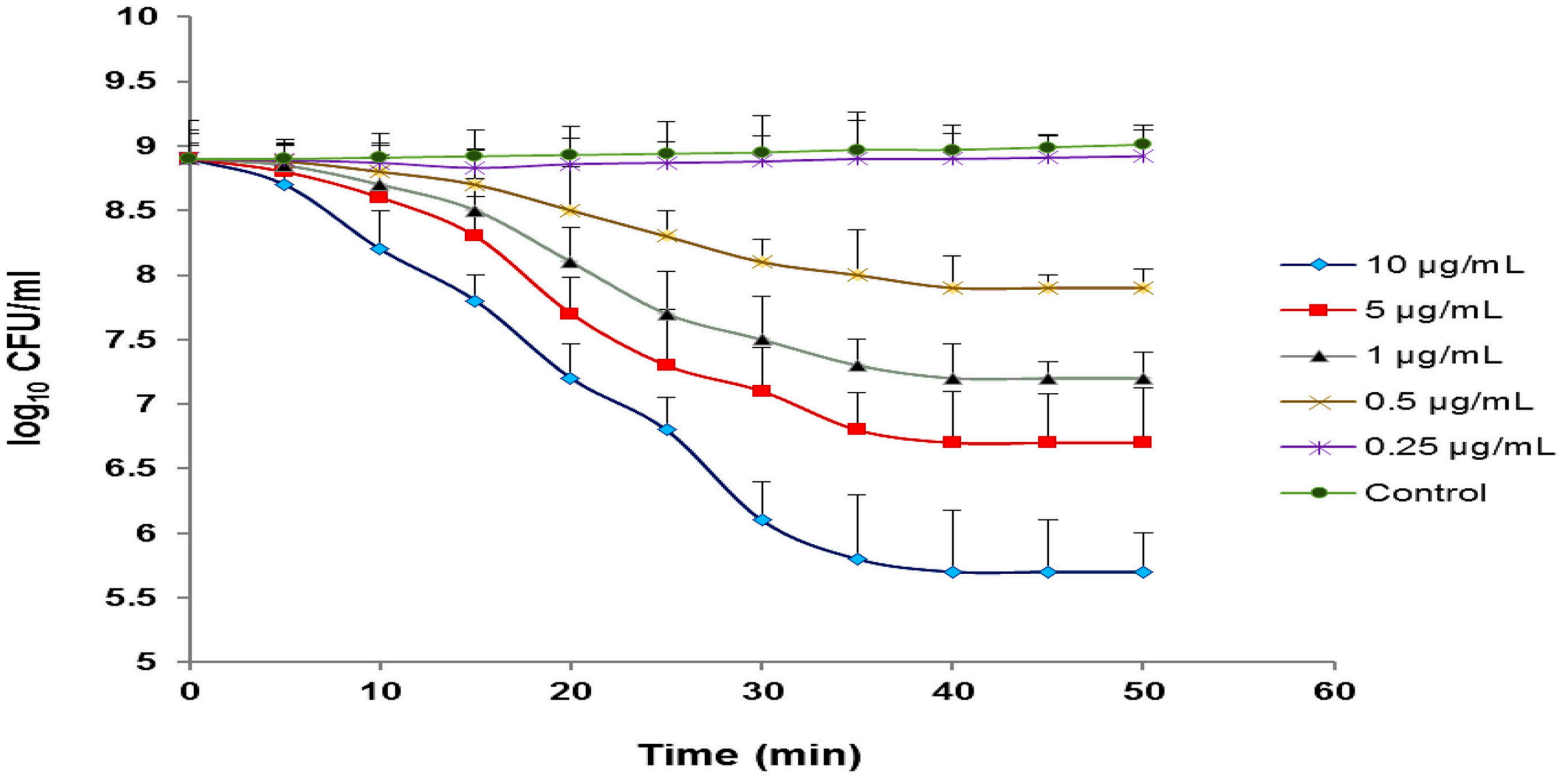
Monitoring the lytic activity of various concentrations of CHAP-amidase against MRSA_252_ cells in MHB medium. The graphs show changes in the colony count of MRSA_252_ cells during a 60-min incubation time. The lysin buffer, rather than the enzyme, was added to negative control. Values are the means of three independent experiments with standard deviation indicated by error bars. Statistical significance was reported as *P* < 0.05.

Our observations revealed that different concentrations of CHAP-amidase, including 1, 5, and 10 μg/mL, can reduce MRSA_252_ titer from 8 × 10^8^ CFU/mL to 1.6 × 10^7^ CFU/mL, 5.1 × 10^6^ CFU/mL and 5.1 × 10^5^ CFU/mL (3.2 log reduction), respectively during 50-min incubation at 37°C (*P* < 0.05). The results indicated that 1 μg/mL is the lowest concentration of CHAP-amidase that is able to inhibit the growth of MRSA_252_ cells (*P* = 0.017). Also, we found that lower concentrations do not have significant effects on the reduction of MRSA turbidity (Figure 8). All assays were performed in triplicate, and data are expressed as the mean ± SD.

### Synergistic interaction between CHAP-amidase and vancomycin

**Figure 9:**
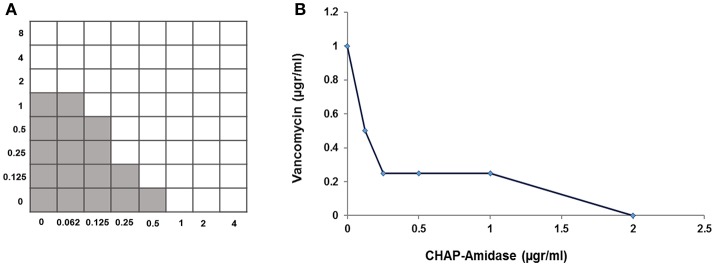
Analysis of synergistic interaction between CHAP-amidase and vancomycin. **(A)** The checkerboard broth microdilution assay of interaction between CHAP-amidase and vancomycin. **(B)** Plotting MICs fractional concentrations along the inhibitory line for enzyme or antibiotic to draw an isobologram (ΣFIC < 0.5).

A standard checkerboard broth micro-dilution assay was used to test whether there is a synergistic interaction between CHAP-amidase and vancomycin. The MIC of CHAP-amidase for MRSA_252_ was determined to be 1 μg/mL, while the MIC of vancomycin for MRSA_252_ was shown to be 2 μg/mL. The enzyme concentrations were transcribed along the inhibitory line on the microtiter plate to draw isobologram (an x/y plot). The highly synergistic interactions were designated to the curve shape for the antibiotic (Figure 9) and were confirmed by the calculation of ΣFIC, which was equal to 0.375.

### Activity of CHAP-amidase endolysin against other bacteria

**Figure 10:**
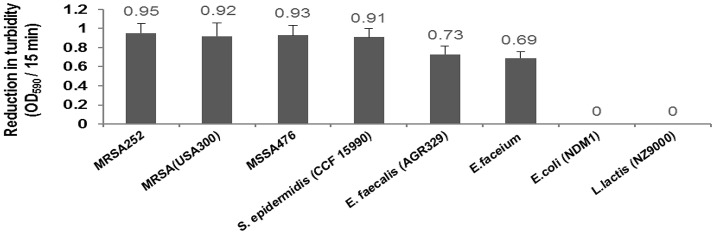
Lytic activity of CHAP-amidase endolysin (10 μg/mL) against different bacterial species. Data are represented as the mean ± SD.

Our results demonstrated CHAP-amidase endolysin could yield strong turbidity reduction and loss of viable plate counts when used against different species of *Staphylococcus* and *Enterococcus*. We observed no significant lytic activity of CHAP-amidase on Gram-negative *E. coli* and Gram positive *Lactococcus lactis* (Figure 10).

## Discussion

In the current study, we proposed *in silico* analysis, expression, purification, and anti-MRSA activity of a novel chimeric protein, which contains CHAP and amidase domains of LysK endolysin. The production of this endolysin has been carried out previously (Horgan et al., 2009; Kashani and Moniri, 2015). However, the current work is the first report on the design and synthesis of specific sequence of CHAP-amidase. The construct we designed was able to produce the small enzymatic protein with proper solubility and activity. The 6x His tag flanked by glycine and serine, which was inserted between the two catalytic domains of the construct, can act as a fusion linker with the ability to prevent spatial interaction of catalytic domains. This linker plays two major roles: poly-His tag sequence may be used for the specific protein detection and GS sequence that is located between CHAP and amidase domains can act as a fusion linker, thereby representing potential to prevent the possibly negative functional interactions between the domains. Glycine and serine residues are known as neutral amino acids and their presence between domains could contribute to the better function of individual domains. Therefore, the linker sequence may lead to an appropriate folding, according to the results obtained from *in silico* analysis. This new chimeric sequence, which consists of 260 amino acids, was successfully produced as a soluble protein. Suitability of the designed protein sequence was analyzed and verified by bioinformatic approaches. The prediction of secondary and tertiary structures of the protein reinforced the important role played by GS/6xHis/GS linker in the design of bifunctional CHAP-amidase protein. Furthermore, Ramachandran plots analyses provided support for the suitability and sustainability of constructed models in which more than 90% of residues were in favored and sterically-allowed regions. These data offer convincing evidence for the protein functionality.

We predicted the binding energy of piperazine, cysteine, and glycerol to CHAP-amidase by using bioinformatics method. The results of our analyses indicated that piperazine and cysteine bind stronger than glycerol to CAHP-amidase. Recent studies have revealed the molecular and crystal structures of CHAP-amidase, as well as some ligands related to this protein (Sanz-Gaitero et al., 2014). Of note, a number of studies have shown that the activity of some antibacterial agents may be enhanced when combined with piperazine and cysteine derivatives (Jack et al., 1995; Andersson and MacGowan, 2003; Nowakowska, 2007).

The recombinant lysins of *S. aureus* have been reported to yield low expression and insolubility when produced in a heterologous host (O'flaherty et al., 2005). However, the chimeric versions of these lysins have exhibited higher expression and activity (Becker et al., 2009; Manoharadas et al., 2009; Daniel et al., 2010). To solve the problem of insolubility, we employed pET vectors containing the N-terminal pelB secretion signal. The use of this signal sequence is considered an ideal approach to direct protein expression to the *E. coli* periplasm (Yoon et al., 2010). Disulfide oxidoreductases and isomerases, which are located in the *E. coli* periplasm, catalyze the proper folding of soluble proteins. This makes the periplasm an appropriate place for the expression of specific therapeutic proteins (Berkmen, 2012). Previous studies have indicated that the exploitation of pET-21 vector may lead to the production of inclusion bodies inside the cytoplasmic area (Sass and Bierbaum, 2007; Abaev et al., 2013). Based on this, we used pET-22b vector for the cloning and expression of LysK-CHAP/amidase. PelB leader sequence in pET-22b vector directs protein secretion to the periplasmic area and also prevents the formation of inclusion bodies in the cytosol, thereby leading to higher expression of soluble proteins. As shown in the results of SDS-PAGE (Figure 5B), we obtained significant protein expression from the soluble phase of *E. coli* lysate. Increased solubility may enhance the activity and improve the function of proteins.

The purified protein indicated lytic inhibitory zone against MRSA_252_ at different concentrations. This finding highlights the antibacterial activity of this chimeric endolysin. The investigation of activity retention strongly suggested that the protein maintains proper folding and is stable under a diverse range of temperatures from 5 to 50°C. Also, tolerating different pH values gives the protein a specific active state. Although, the protein remained active at various salt concentrations, it showed the highest activity in the absence of NaCl. The results of the current study also highlighted that our recombinant endolysin shows more potent activity (at 10 and 5 μg/mL concentration) against MRSA_252_ compared with previously studied endolysin constructs (Becker et al., 2009; Fernandes et al., 2012; Proença et al., 2012).

Our *in vitro* studies demonstrated the presence of a synergistic interaction between chimeric CHAP-amidase and vancomycin as a commonly used antibiotic to treat staphylococcal infections (Daniel et al., 2010). Isobolographic analysis confirmed this synergistic effect against MRSA_252_ strain with FIC Index of 0.39. This finding is consistent with previous studies that have indicated a synergistic interplay between some regular antibiotics and chimeric endolysins (Manoharadas et al., 2009; Daniel et al., 2010). Formerly, it has been revealed that native LysK exhibits strong lytic activity against a wide range of staphylococcal species, but it does not show any lytic activity against other bacterial groups (O'Flaherty et al., 2005). In our work, CHAP-amidase presented potent activity against vancomycin-resistant species of *Enterococcus* wherein the colony counts of *Enterococcus faecalis* and *Enterococcus faecium* reduced from 8 × 10^8^ to about 3 × 10^8^ CFU/mL within a 15-min incubation at 37°C. The endolysin we produced did not show significant antibacterial activity against multi-drug resistant *E. coli* as a representative of Gram-negative bacteria and *Lactococcus lactis* as a prominent probiotic bacterium. Due to rapid activity and high specificity, the chimeric endolysin obtained in our study could have important implications for the treatment of bacterial infections.

## Conclusion

The results of *in silico* design and analyses suggested the new arrangement of functional CHAP-amidase domains. The purified chimeric protein presented optimal characteristics and specific activity under different thermal, pH and saline conditions. We observed significant antibacterial activity of endolysin against methicillin-resistant *Staphylococcus aureus*, also the evidence of a synergism between CHAP-amidase and vancomycin suggests the possibility to use lower doses of vancomycin against pathogenic *S. aureus* strains. Moreover, we found antibacterial activity of endolysin against some antibiotic-resistant bacteria. All of these findings favor the notion that the chimeric endolysin produced in our work may offer promise for the development of an efficient therapeutic and antimicrobial agent in the future. However, further investigation is required to provide more evidence on different aspects of the antibacterial activity of this endolysin.

## Ethics statement

**Ethical Responsibilities of Authors:** This paper is our original unpublished work and it has not been submitted to any other journal for reviews.

**Compliance with Ethical Standards:** This article does not contain any studies with human participants or animals performed by any of the authors.

## Author contributions

RM and HH have conducted the research work. HH and YD were written the manuscript. HH and HF were analyzed the data.

### Conflict of interest statement

The authors declare that the research was conducted in the absence of any commercial or financial relationships that could be construed as a potential conflict of interest.
