# Supplementary figures and images for: A Novel Chimeric Endolysin with Antibacterial Activity against Methicillin-Resistant Staphylococcus aureus
Source: Front Cell Infect Microbiol. 2017 Jun 30;7:290. doi: 10.3389/fcimb.2017.00290 (PMC5491540; doi:10.3389/fcimb.2017.00290)

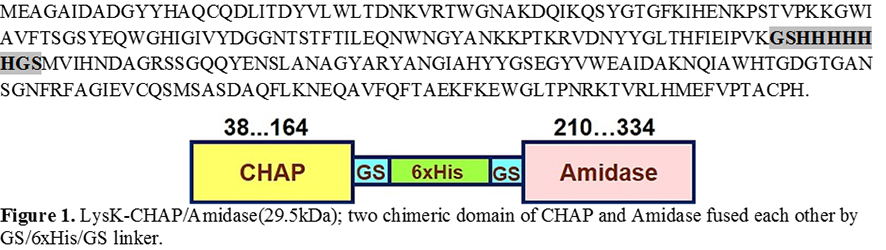

Supplement: Supplementary file 1 [file Image1.TIF]

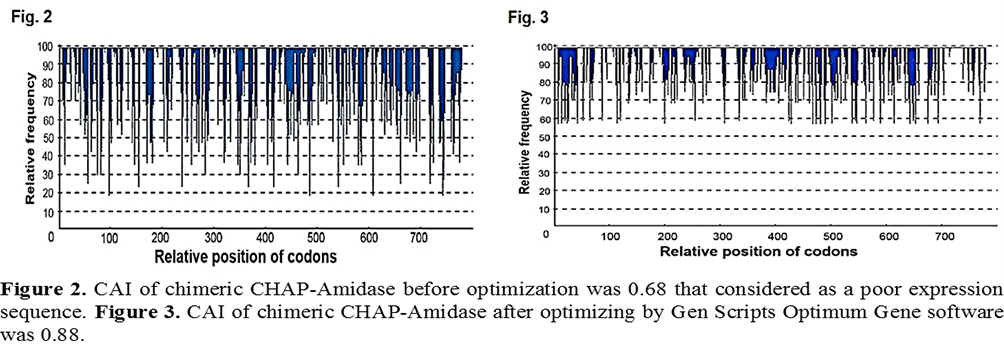

Supplement: Supplementary file 2 [file Image2.TIF]

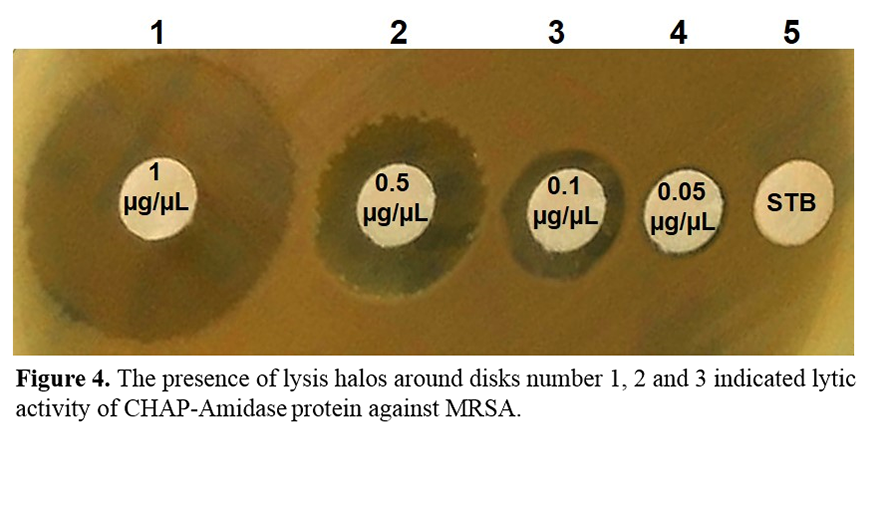

Supplement: Supplementary file 3 [file Image3.TIF]
